# Endovascular treatment of patients with stroke caused by anterior cerebral artery occlusions

**DOI:** 10.1007/s13760-023-02395-8

**Published:** 2023-10-12

**Authors:** Erik M. Vos, Manon Kappelhof, Sanne J. den Hartog, Jonathan M. Coutinho, Bart J. Emmer, Bob Roozenbeek, Wim H. van Zwam, Robert J. van Oostenbrugge, H. Bart van der Worp, Maarten Uyttenboogaart, Adriaan C. G. M. van Es, Charles B. L. M. Majoie, Diederik W. J. Dippel, Cacha M. P. C. D. Peeters-Scholte, Ido R. van den Wijngaard, the MR CLEAN Registry Investigators

**Affiliations:** 1 Department of Neurology, Haaglanden Medical Center, The Hague, The Netherlands; 2 Department of Radiology and Nuclear Medicine, Amsterdam UMC Location University of Amsterdam, Amsterdam, The Netherlands; 3 Department of Neurology, Radiology and Nuclear Medicine, Erasmus Medical Center, Public Health, Rotterdam, The Netherlands; 4 Department of Neurology, Amsterdam UMC Location University of Amsterdam, Amsterdam, The Netherlands; 5 Department of Radiology and Nuclear Medicine, School for Cardiovascular Diseases (CARIM), Maastricht UMC+, Maastricht, The Netherlands; 6 Department of Neurology, School for Cardiovascular Diseases (CARIM), School for Mental Health and Neuroscience, Maastricht UMC+, Maastricht, The Netherlands; 7 Department of Neurology and Neurosurgery, Brain Center, University Medical Center Utrecht, Utrecht, The Netherlands; 8 Department of Neurology, Department of Radiology, Medical Imaging Center, University Medical Center Groningen, Groningen, The Netherlands; 9 Department of Radiology, Leiden University Medical Center, Leiden, The Netherlands; 10 Department of Neurology, Erasmus Medical Center, Rotterdam, The Netherlands; 11 Department of Neurology, Leiden University Medical Center, Leiden, The Netherlands

## Abstract

**Background:**

Occlusion of the anterior cerebral artery (ACA) is uncommon but may lead to significant disability. The benefit of endovascular treatment (EVT) for ACA occlusions remains uncertain.

**Methods:**

We included patients treated with EVT and compared patients with ACA occlusions with patients who had internal carotid artery (ICA) or proximal (M1/M2) middle cerebral artery (MCA) occlusions from the MR CLEAN Registry. Primary outcome was the modified Rankin Scale score (mRS). Secondary outcomes were functional independence (mRS 0–2), National Institutes of Health Stroke Scale (NIHSS) score, delta-NIHSS (baseline minus NIHSS score at 24–48 h), and successful recanalization (expanded thrombolysis in cerebral infarction (eTICI) score 2b-3). Safety outcomes were symptomatic intracranial hemorrhage (sICH), periprocedural complications, and mortality.

**Results:**

Of 5193 patients, 11 (0.2%) had primary ACA occlusions. Median NIHSS at baseline was lower in patients with ACA versus ICA/MCA occlusions (11, IQR 9–14; versus 15, IQR 11–19). Functional outcome did not differ from patients with ICA/MCA occlusions. Functional independence was 4/11 (36%) in patients with ACA versus 1949/4815 (41%) in ICA/MCA occlusions; median delta-NIHSS was − 1 (IQR − 7 to 2) and − 4 (IQR − 9 to 0), respectively. Successful recanalization was 4/9 (44%), versus 3083/4787 (64%) in ICA/MCA occlusions. Mortality was 3/11 (27%) versus 1263/4815 (26%). One patient with ACA occlusion had sICH; no other complications occurred.

**Conclusion:**

In this cohort ACA occlusions were uncommon. Functional outcome did not differ between patients with ACA occlusions and ICA/MCA occlusions. Prospective research is needed to determine feasibility, safety, and outcomes of EVT for ACA occlusions.

## Introduction

Acute ischemic stroke (AIS) as a result of isolated primary occlusion of the anterior cerebral artery (ACA) accounts for only 0.5–3% of all strokes [1] but may lead to significant handicap. Patients typically present with contralateral weakness of the leg and may suffer cognitive deficits and behavioral changes due to ischemia in limbic and frontal areas of the brain [2]. Occasionally, contralateral weakness of the face and arm is present when the medial lenticulostriate arteries are involved[3]. Since the publication of five large trials in 2015, [4–8] endovascular treatment (EVT) has become standard care for AIS due to proximal anterior circulation occlusions[9]. However, the majority of patients in the EVT trials had an internal carotid artery (ICA) or proximal middle cerebral artery (MCA) occlusion. Of all 5 trials, only the Multicenter Randomized Clinical trial of Endovascular treatment for Acute ischemic stroke in the Netherlands (MR CLEAN) allowed inclusion of patients with ACA occlusions, and only three such patients were included[4]. As such, the benefit of EVT for ACA occlusions remains uncertain and data on treatment outcomes in clinical practice are scarce. Observational data suggest that EVT for these patients is safe and feasible, but the reported number of patients is limited [10–13]. Current guidelines do not provide clear recommendations for ACA occlusions and treatment for these patients varies between countries, centers, and interventionalists[14]. The aim of this study was to describe the results and outcome of EVT in patients with primary ACA occlusions using a large Dutch national EVT registry [15].

## Methods

### Study design

The MR CLEAN Registry is a multicenter, nationwide, prospective, consecutive database of patients treated with EVT for AIS in the Netherlands. Patients were included if they underwent arterial puncture with the intention to perform EVT for AIS. The choice of EVT technique and device was left to the treating interventionalist. Radiological baseline and follow-up imaging were centrally adjudicated by an independent core laboratory of trained physicians blinded to all clinical and treatment data except for symptom side. The MR CLEAN Registry study protocol was evaluated by the ethics committee of the Erasmus MC, University Medical Centre, Rotterdam, the Netherlands (MEC-2014-235). Permission to carry out the study as a registry was granted.

We used data of patients treated between March 16, 2014, from the first patient treated after the MR CLEAN trial [4], and January 1, 2019. We compared patients with ACA occlusions on computed tomography angiography (CTA), aged 18 years or older, who were treated in a MR CLEAN trial center, with patients with occlusion of the ICA or proximal MCA (M1 and M2 parts). Patients with secondary (periprocedural) occlusion of the ACA or additional occlusions in different vascular territories were excluded.

### Outcome measures

Our primary outcome was functional outcome measured on the modified Rankin Scale (mRS, ranging from 0, no symptoms, to 6, death) at 90 days after stroke onset. Secondary clinical and radiological outcomes were 90-day functional independence (mRS 0–2), National Institutes of Health Stroke Scale (NIHSS) score at 24–48 h, delta-NIHSS (baseline NIHSS minus NIHSS score at 24–48 h), NIHSS improvement ($\ge$ 4 NIHSS points improvement between baseline and 24–48 h), and successful reperfusion (expanded thrombolysis in cerebral infarction (eTICI) score 2b–3). Safety outcomes were 90-day mortality, symptomatic intracranial hemorrhage (sICH), peri-operative complications (vessel perforation, dissection), and neurological deterioration ($\ge$ 4 NIHSS points worsening between baseline and 24–48 h).

### Statistical analysis

All analyses were descriptive because of the low number of patients with ACA occlusions. Summary statistics are presented as mean with standard deviation for normally distributed data, and median with interquartile range (IQR) for non-normally distributed data. We compared outcomes of patients with ACA occlusions with those of MR CLEAN Registry patients with ICA or proximal MCA occlusions. For binary categorical outcomes, Fisher’s exact test was used in case of expected counts of zero and Chi-Square test if there were no expected counts of zero. For ordinal outcomes, the Chi-Square test for trend was used. For the semi-continuous outcome measure of delta-NIHSS, which was non-normally distributed, the Kruskal–Wallis test was used. Missing data were not imputed. The level of significance was set at *p* < 0.05. All statistical analyses were performed with IBM SPSS Statistics version 23.0.0.0 (New York, NY, United States 2017).

### Data availability

In compliance with the General Data Protection Regulation, source data will not be made available for other researchers. Analytic methods, study materials, scripts of the statistical analyses, and their output are available from the corresponding author on reasonable request.

## Results

**Fig. 1:**
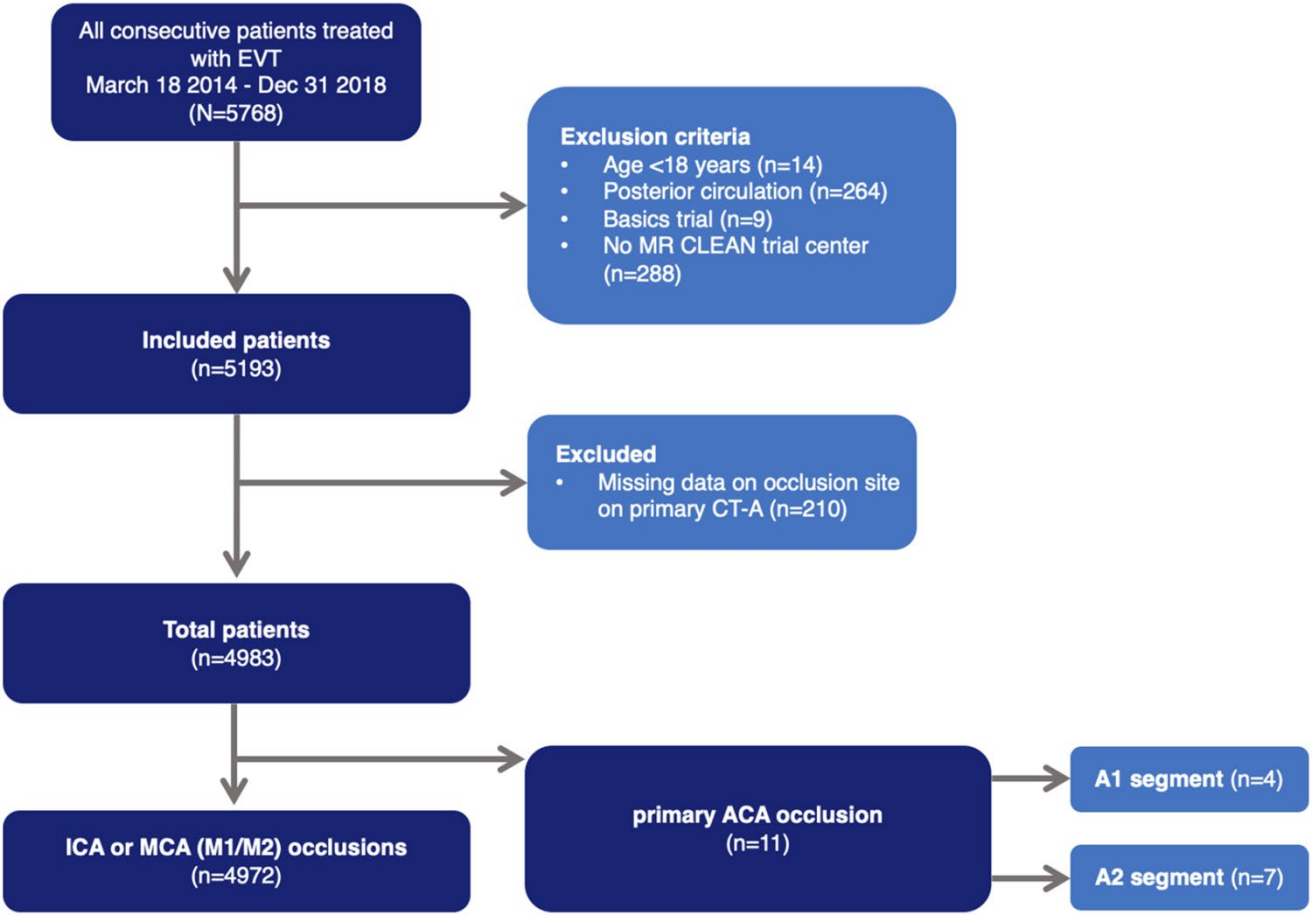
Patient inclusion flow chart A1, first segment of anterior cerebral artery; A2, second segment of anterior cerebral artery; ACA, anterior cerebral artery; CTA, computed tomography angiography; MCA, middle cerebral artery; M1, first segment of middle cerebral artery; M2, second segment of middle cerebral artery; MR CLEAN, Multicenter Randomized CLinical trial of Endovascular treatment for Acute ischemic stroke in the Netherlands

In total, 5193 patients were included in the MR CLEAN Registry between March 16, 2014 and January 1, 2019. Data on the site of occlusion on baseline CTA were missing for 210/5193 (4.0%) patients. Only 11 patients (0.2%) had a primary ACA occlusion on CTA at admission. Four out of 11 patients had a occlusion in the A1 segment (36%) and seven patients in the A2 segment (64%) (Fig. 1). In patients with ACA occlusions, median age was 69 (IQR 65–74 years) versus 72 (IQR 62–81) in patients with ICA or MCA occlusions. Three (27%) patients with ACA occlusions were female versus 2396 (48%) in the ICA/MCA group. Median NIHSS score on admission was lower in patients with ACA occlusions (11, IQR 9–14) compared to patients with ICA or MCA occlusions (15, IQR 11–19; Table 1).

**Table 1:** Demographic variables

	Patients with primary ACA occlusions (*n* = 11)	Missing	Patients with ICA or MCA (M1/2) occlusions (*n* = 4972)	Missing
Clinical characteristics				
Age (years); median (IQR)	69 (65–74)	0	72 (62–81)	0
Female: *n* (%)	3 (27)	0	2396 (48)	0
Systolic blood pressure (mmHg); mean (SD)	139 (29)	0	150 (26)	142
NIHSS score; median (IQR)	11 (9–14)	2	15 (11–19)	67
Intravenous thrombolysis: n (%)	5 (46)	0	3435 (69)	8
Pre-stroke mRS; *n* (%)		2		129
mRS 0	6 (55)		3141 (65)	
mRS 1	0 (0)		692 (14)	
mRS ≥ 2	3 (27)		1010 (21)	
Medical history; *n* (%)				
Previous stroke	3 (27)	0	874 (18)	39
Previous myocardial infarction	3 (27)	0	696 (14)	92
Previous peripheral artery disease	1 (9)	0	454 (9)	92
Hypertension	6 (55)	0	2590 (53)	95
Hypercholesterolemia	4 (36)	0	1476 (31)	204
Atrial fibrillation	2 (18)	1	1187 (24)	66
Diabetes	2 (18)	0	824 (17)	30
Smoking	3 (27)	0	1037 (21)	49
Antiplatelet therapy	4 (36)	1	1532 (31)	58
DOAC/Coumarin use	1 (9)	0	859 (18)	96
Occlusion location; *n* (%)		0		0
A1 segment	4 (36)		–	
A2 segment	7 (64)		–	
ICA-T	–		958 (19)	
Proximal M1	–		1222 (25)	
Distal M1	–		1558 (31)	
Intracranial ICA	–		266 (5)	
M2	–		943 (19)	
Median process times (min); median (IQR)				
Door to Needle time—IVT	31 (24–50)	6	24 (18–33)	2216
Door to Groin time—EVT	79 (54–114)	3	57 (34–86)	461
Onset to reperfusion time	250 (177–303)	1	250 (192–322)	518
EVT procedure duration	52 (36–82)	2	55 (35–80)	592

*DOAC* direct oral anticoagulant, *EVT* endovascular treatment, *ICA(-T)* internal carotid artery (-terminus), *IQR* interquartile range, *IVT* intravenous thrombolytic therapy, *A1* first segment of anterior cerebral artery, *A2* second segment of anterior cerebral artery, *M1* first segment of middle cerebral artery, *M2* second segment of middle cerebral artery, *mRS* modified Rankin Scale score, *MR CLEAN* Multicenter Randomized CLinical trial of Endovascular treatment for Acute ischemic stroke in the Netherlands, *NIHSS* National Institutes of Health Stroke Scale

### Functional outcomes

**Fig. 2:**
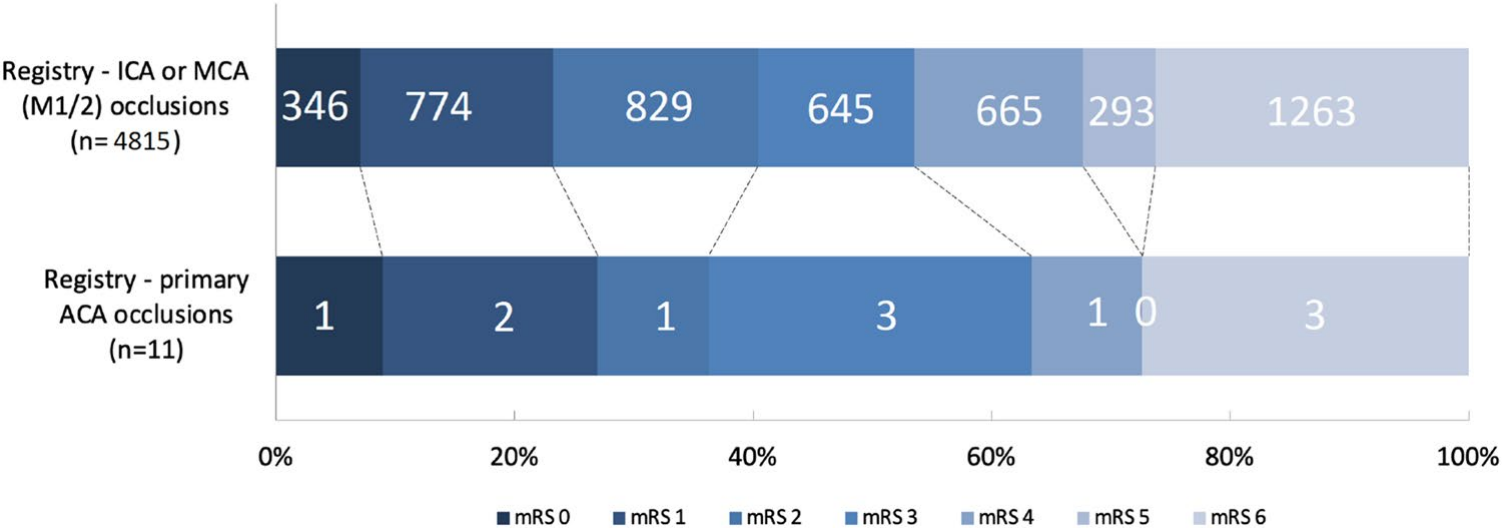
Ordinal mRS outcomes in patients with ACA occlusions versus ICA or MCA (M1/2) occlusions in the MR CLEAN Registry *ACA* anterior cerebral artery, *mRS* modified Rankin Scale score

Functional outcome at 90 days after AIS did not differ between patients with ACA occlusions and those with ICA or MCA occlusions (Fig. 2). Four of 11 patients (36%) with ACA occlusions were functionally independent at 3 months, versus 1949/4815 (41%) patients with.

ICA or MCA occlusions (Table 2). In patients with ACA occlusions, median NIHSS score at 24–48 h after AIS onset was 11 (IQR 2–13) with a median delta-NIHSS of -1 (IQR -7 to 2). In patients with ICA or proximal MCA occlusions, median NIHSS score at 24–48 h after AIS onset was 9 (IQR 3–17) with a median delta-NIHSS of -4 (IQR -9 to 0). Three of 9 patients (33%; 2 missing data) in the ACA group showed improvement of ≥ 4 points on the NIHSS versus 2427/4518 patients (54%) in the ICA or MCA group.

**Table 2:** Results IPO-ACA versus all other occlusions

Outcome	Patients with IPO-ACA total (*n* = 11)	Patients with ICA or MCA (M1/2) occlusions (*n* = 4972)
Functional outcomes		
mRS at 90 days; median (IQR)*	3 (1–6)	3 (2–6)
Functional independence (mRS 0–2), *n* (%)	4/11 (36)	1949/4815 (41)
NIHSS 24–48 h after stroke; median (IQR)†	11 (2–13)	9 (3–17)
Delta-NIHSS; median (IQR)‡	–1 (–7 to 2)	–4 (–9 to 0)
NIHSS improvement ≥ 4 points; n (%)	3/9 (33)	2427/4518 (54)
Radiological outcomes		
eTICI score; n (%) §		
0	3 (33)	749 (16)
1	0 (0)	121 (3)
2A	2 (22)	825 (17)
2B	1 (11)	1082 (23)
2C	0 (0)	520 (11)
3	3 (33)	1481 (31)
Successful reperfusion (TICI 2B-3)	4 (44)	3083 (64)
Safety outcomes		
Periprocedural perforation; *n* (%) ¶	0/9 (0)	65/4318 (2)
Periprocedural dissection; *n* (%) ††	0/9 (0)	77/4330 (2)
Symptomatic intracranial hemorrhage; *n* (%)	1/11 (9)	294/4972 (6)
NIHSS deterioration ≥ 4 points; *n* (%) §§	1/9 (11)	532/4518 (12)
Mortality at 90 days; *n* (%)	3/11 (27)	1263/4815 (26)

* missing: 157; † missing: 436; ‡ missing: 456; § missing: 196; ¶ missing: 656; †† missing: 644; §§ missing: 454. Change in NIHSS from baseline to 24–48 h after stroke. *ACA* anterior cerebral artery, *eTICI* expanded thrombolysis in cerebral Infarction, *IPO-ACA*, isolated primary occlusion of the anterior cerebral artery, *IQR*, interquartile range, *mRS*, modified Rankin Scale, *MR CLEAN*, Multicenter Randomized CLinical trial of Endovascular treatment for Acute ischemic stroke in the Netherlands, *NIHSS*, National Institutes of Health Stroke Scale

### Radiological outcomes

Successful recanalization was achieved in 4/9 (44%) of patients with ACA occlusions (Table 2). Of these patients, 2/4 (50%) were functionally independent at 3 months versus 2/5 (40%) of patients with partial or no recanalization. Successful recanalization was achieved in 3083/4787 (64%) of patients with ICA or proximal MCA occlusions. Of these patients, 1451/2977 (49%) achieved functional independence at 3 months versus 418/1656 (25%) of patients with partial or no recanalization. Procedural characteristics are shown in Table 3.

**Table 3:** Procedure characteristics in treated ACA strokes

	Occlusion site (CTA)	Occlusion site (DSA)	Baseline ASPECTS	Collateral score	Description of procedure	Intervention	Post treatment eTICI score
1	Right A1	Right A1	7	3	No EVT possible due to elongated carotid arteries, full recovery after IVT	IVT	*Not available*
2	Left A1	Left A1	8	1	8F guiding catheter, Solitaire stent retriever	EVT + IVT	3
3	Right A2	Right A2	10	2	6F guiding catheter, TREVO stent retriever	EVT	2B
4	Left A1	*Not* *available*	10	2	6F guiding catheter, tortuous anatomy, no navigation to A1 possible	DSA only	1
5	Right A2	Right A2	8	2	6F guiding catheter, carotid: floating thrombus from stenosis/web. Double MCA. No real occlusion in MCA. Anterior branch A2 occluded, not treated	DSA only + IVT	1
6	Right A1	Right A2	10	3	6F guiding catheter, TREVO Stent-retriever	EVT + IVT	3
7	Left A2	Left A3	8	2	6F guiding catheter, TREVO Stent-retriever	EVT	2A
8	Right A2	Right A1	10	2	6F guiding catheter, TREVO Stent-retriever, intracranial dissection A2, stent placed, re-occluded	EVT + Stent	1
9	Left A1	*Not* *available*	10	3	6F guiding catheter	Catheterization only	1
10	A3	*Not* *available*	7	1	8F guiding catheter, aspiration with penumbra	EVT + IVT	3
11	Left A2	Left A2	9	3	Aspiration with penumbra Ace 68, information on guiding catheter not available	EVT	3

*ACA* anterior cerebral artery, *ASPECTS* Alberta Stroke Program Early CT Score, Collateral score, 0: absent, 1: poor, 2: moderate, 3: good; *EVT* endovascular treatment, *IVT* intravenous thrombolysis

### Safety outcomes

Mortality at 90 days was 27% (3/11) in the ACA group versus 26% (1263/4815) in patients with ICA or MCA occlusions (Table 2). One of 11 patients (9%) in the ACA group had sICH, and one patient (9%) deteriorated ≥ 4 points on the NIHSS. No periprocedural dissection or perforation occurred in this group. In patients with ICA or proximal MCA occlusions, 294/4972 (6%) had sICH, periprocedural dissection occurred in 77/4330 (2%), and 65/4318 (2%) suffered peri-procedural perforation.

## Discussion

In this Dutch national cohort of EVT-treated patients, ACA occlusions were uncommon, occurring in only 0.2% of 4983 patients. Complication rates and functional outcomes of patients with ACA occlusions were similar to those of patients with ICA or proximal MCA occlusions. However, analyses were limited by the small ACA sample size. Successful reperfusion was achieved in less than half of patients with ACA occlusions.

Even though ACA occlusions were an inclusion criterion in the MR CLEAN trial, less than 1% of patients in the subsequent MR CLEAN Registry were treated for ACA occlusions. As ACA occlusions may account for up to 3% of all stroke cases, [1] this suggests under-treatment and possibly under-recognition of ACA occlusions. The uncertainty of the available evidence and lack of consensus on the benefit of EVT for ACA occlusions in stroke may have negatively impacted the number of patients with ACA occlusions treated with EVT in our study. ACA occlusions in stroke may be more likely to be missed on baseline CTA, as the acute diagnostic workup for stroke patients typically focusses on the MCA branches [16]. In addition, due to variations in ACA anatomy and collateral blood flow, ACA strokes are often accompanied by non-specific symptoms and ACA occlusions may initially lead to little or no recognized sequelae [2, 3]. Careful evaluation of the ACA on imaging is warranted in all patients with stroke-like symptoms, specifically those presenting with disproportionate lower extremity weakness. Useful sequences for evaluating the ACA are maximum intensity projections—specifically the sagittal reconstructions—as well as multiphase CTA and/or CT Perfusion imaging (CTP) [3]. In recent years the increasing use of CTP has improved detection of large and medium vessel occlusions (LVO, MeVO) [10]. This may have led to increased detection and EVT of ACA occlusions in more recent years.

Data on outcomes of EVT in patients with ACA occlusion are scarce. Most studies are retrospective and include a small number of patients, even when large patient databases are used [11–13]. Despite high reported percentages of successful recanalization, several studies report functional independence rates after treating ACA occlusions of less than 40% [11–13, 17]. As ACA occlusions tend to be classified as MeVO [18], some studies on outcomes in EVT-treated stroke patients with MeVO also included ACA occlusions [19, 20]. Several trials on EVT in MeVO are currently ongoing, most notably EndovaSCular TreAtment to imProve outcomEs for Medium Vessel Occlusions (ESCAPE-MeVO, NCT05151172), Distal Ischemic Stroke Treatment With Adjustable Low-profile Stentriever (DISTALS, NCT05152524), EnDovascular Therapy Plus Best Medical Treatment (BMT) Versus BMT Alone for MedIum VeSsel Occlusion sTroke (DISTAL, NCT05029414), and Evaluation of Mechanical Thrombectomy in Acute Ischemic Stroke Related to a Distal Arterial Occlusion (DISCOUNT, NCT05030142). However, the inclusion of other types of MeVO such as MCA- M2-4 occlusions, ACP-P1-2, and PICA and AICA occlusions may affect the evidence on benefit of EVT for ACA occlusions. Most observational studies do not differentiate between primary ACA and secondary ACA occlusions (e.g., iatrogenic after intravenous thrombolysis or EVT) [3, 20]. In our study, the rate of successful reperfusion after EVT for primary ACA occlusions was only 44%, which is lower compared to other reported series (73–100%), though our rate of functional independence was in line with previous studies [11–13, 17, 21].

Technically, endovascular procedures in the ACA are more challenging compared to the MCA territory. Technical difficulty increases with more distal occlusions (i.e., A2/A3 compared to A1). Catheterizing the ACA from the distal ICA is more difficult due to its curved anatomy and interventionalists need to avoid crossing the anterior communicating artery. The lower recanalization percentage in this study may be explained by the high number of patients with A2 occlusions (64%) in our cohort. The high number of patients with A2 occlusions may also explain the significantly lower NIHSS scores in patients with ACA occlusions at baseline.

Our study has limitations. First, the number of included patients with ACA occlusions was small, limiting the power and reliability of the comparisons made. The small group size also made further statistical modeling with covariate adjustment unfeasible. Second, the MR CLEAN Registry only included EVT-treated patients, so true treatment benefit of EVT compared to best medical care only could not be determined. ACA occlusions may occur more often, but may not be not treated with EVT in the absence of proven treatment benefit and clear guidelines, after which they would not be included in our current data set. Evaluation of consecutive EVT-treated and non-EVT-treated patients with ACA occlusions would be of great value. Third, the eTICI score used in the MR CLEAN Registry may be suboptimal in measuring the benefit of EVT in patients with ACA occlusions. The eTICI score is mostly used for assessing the MCA territory. As such, interpretation and scoring of the ACA territory may be subject to higher interobserver variability. Lastly, the data in our cohort span a period of several years, starting at the beginning of the widespread use of EVT in treating AIS. In recent years, EVT techniques have improved by using smaller or adjustable stent retrievers, increasingly more experienced interventionalists, and ongoing research [22]. Consequently, current interventions may actually lead to better recanalization and functional outcomes in patients with ACA occlusions [22]. The recent introduction of new generation small caliber catheters and low-profile stent retrievers has allowed access to more distal sites of ACA. The effect on outcome of EVT for distal (ACA) occlusions is currently being investigated in several clinical trials, such as DISTALS and DISCOUNT [23]. More data and analyses of existing data are needed to determine the benefit and safety of EVT in patients with ACA occlusions. Ideally, these data should be prospective in order to gather high-quality evidence on this topic.

## Conclusion

In this Dutch national cohort of EVT-treated patients, patients with ACA occlusions were uncommon, occurring in only 0.2% of 4983 patients. Complication rates and functional outcomes were similar to those of EVT-treated patients with ICA and proximal MCA occlusions, though analyses were limited by the small ACA occlusion group sample size. Successful reperfusion was achieved in less than half of patients with ACA occlusions. These data may suggest that EVT is safe in ACA stroke, though more technically challenging. Prospective research is needed to confirm data on feasibility, safety, and outcomes of EVT for ACA strokes.

## Disclosures

CBLMM received grants from CVON/Dutch Heart Foundation, Stryker, European Commission, TWIN, and Dutch Health Evaluation Program) and is a shareholder of Nico.Lab. DWJD received grants from Dutch Heart Foundation, Brain Foundation Netherlands, The Netherlands Organisation for Health Research and Development, Health Holland Top Sector Life Sciences & Health, Stryker, Penumbra Inc., Medtronic, Thrombolytic Science LLC, and Ceronovus. JMC received grants from Boehringer Ingelheim, Bayer, and Medtronic. PJvD received support from Stryker. WHvZ received personal support from Cerenovus, Stryker, Nicolab, and Philips. CPS received support from Neurophyxia BV. HBvdW received funding for consultancy from Bayer and TargED, and was paid to his institution. Others disclose nothing. MU received grants from Dutch Heart Foundation.

## Appendix

MR CLEAN Registry investigators:

Executive committee

Diederik W.J. Dippel^1^; Aad van der Lugt^2^; Charles B.L.M. Majoie^3^; Yvo B.W.E.M. Roos^4^; Robert J. van Oostenbrugge^5,44^; Wim H. van Zwam^6,44^; Jelis Boiten^14^; Jan Albert Vos^8^

Study coordinators

Ivo G.H. Jansen^3^; Maxim J.H.L. Mulder^1,2^; Robert- Jan B. Goldhoorn^5,6,44^; Kars C.J. Compagne^2^; Manon Kappelhof^3^; Josje Brouwer^4^; Sanne J. den Hartog^1,2,40^; Wouter H. Hinsenveld ^5,6^; Lotte van den Heuvel^1,40^.

Local principal investigators

Diederik W.J. Dippel^1^; Bob Roozenbeek^1^; Aad van der Lugt^2^; Pieter Jan van Doormaal^2^, Charles B.L.M. Majoie^3^; Yvo B.W.E.M. Roos^4^; Bart J. Emmer^3^; Jonathan M. Coutinho^4^; Wouter J. Schonewille^7^; Jan Albert Vos^8^; Marieke J.H. Wermer^9^; Marianne A.A. van Walderveen^10^; Adriaan C.G.M. van Es^10^; Julie Staals^5,44^; Robert J. van Oostenbrugge^5,44^; Wim H. van Zwam^6,44^; Jeannette Hofmeijer^11^; Jasper M. Martens^12^; Geert J. Lycklama à Nijeholt^13^; Jelis Boiten^14^; Sebastiaan F. de Bruijn^15^; Lukas C. van Dijk^16^; H. Bart van der Worp^17^; Rob H. Lo^18^; Ewoud J. van Dijk^19^; Hieronymus D. Boogaarts^20^; J. de Vries^22^; Paul L.M. de Kort^21^; Julia van Tuijl^21^; Issam Boukrab^26^; Jo P. Peluso^26^; Puck Fransen^22^; Jan S.P. van den Berg^22^; Heleen M. den Hertog^22^; Boudewijn A.A.M. van Hasselt^23^; Leo A.M. Aerden^24^; René J. Dallinga^25^; Maarten Uyttenboogaart^28^; Omid Eschgi^29^; Reinoud P.H. Bokkers^29^; Tobien H.C.M.L. Schreuder^30^; Roel J.J. Heijboer^31^; Koos Keizer^32^; Rob A.R. Gons^32^; Lonneke S.F. Yo^33^; Emiel J.C. Sturm^35,47^, Tomas Bulut^35^; Paul J.A.M. Brouwers^34^; Anouk D. Rozeman^42^; Otto Elgersma^41^, Michel J.M. Remmers^43^; Thijs E.A.M. de Jong^46^.

Imaging assessment committee

Charles B.L.M. Majoie^3^(chair); Wim H. van Zwam^6,44^; Aad van der Lugt^2^; Geert J. Lycklama à Nijeholt^13^; Marianne A.A. van Walderveen^10^; Marieke E.S. Sprengers^3^; Sjoerd F.M. Jenniskens^27^; René van den Berg^3^; Albert J. Yoo^38^; Ludo F.M. Beenen^3^; Alida A. Postma^6.45^; Stefan D. Roosendaal^3^; Bas F.W. van der Kallen^13^; Ido R. van den Wijngaard^13^; Adriaan C.G.M. van Es^10^; Bart J. Emmer,^3^; Jasper M. Martens^12^; Lonneke S.F. Yo^33^; Jan Albert Vos^8^; Joost Bot^36^; Pieter-Jan van Doormaal^2^; Anton Meijer^27^; Elyas Ghariq^13^; Reinoud P.H. Bokkers^29^; Marc P. van Proosdij^37^; G. Menno Krietemeijer^33^; Jo P. Peluso^26^; Hieronymus D. Boogaarts^20^; Rob Lo^18^; Wouter Dinkelaar^41^; Auke P.A. Appelman^29^; Bas Hammer^16^; Sjoert Pegge^27^; Anouk van der Hoorn^29^; Saman Vinke^20^; Sandra Cornelissen^2^; Christiaan van der Leij^6^; Rutger Brans^6^; Jeanette Bakker^41^; Maarten Uyttenboogaart^28^; Miou Koopman^3^; Lucas Smagge^2^; Olvert A. Berkhemer^1,3,6^; Jeroen Markenstein^3^; Eef Hendriks^3^; Patrick Brouwer^10^; Dick Gerrits^35^.

Writing committee

Diederik W.J. Dippel^1^(chair); Aad van der Lugt^2^; Charles B.L.M. Majoie^3^; Yvo B.W.E.M. Roos^4^; Robert J. van Oostenbrugge^5,44^; Wim H. van Zwam^6,44^; Geert J. Lycklama à Nijeholt^13^; Jelis Boiten^14^; Jan Albert Vos^8^; Wouter J. Schonewille^7^; Jeannette Hofmeijer^11^; Jasper M. Martens^12^; H. Bart van der Worp^17^; Rob H. Lo^18^

Adverse event committee

Robert J. van Oostenbrugge^5,44^(chair); Jeannette Hofmeijer^11^; H. Zwenneke Flach^23^

Trial methodologist

Hester F. Lingsma^40^

Research nurses / local trial coordinators

Naziha el Ghannouti^1^; Martin Sterrenberg^1^; Wilma Pellikaan^7^; Rita Sprengers^4^; Marjan Elfrink^11^; Michelle Simons^11^; Marjolein Vossers^12^; Joke de Meris^14^; Tamara Vermeulen^14^; Annet Geerlings^19^; Gina van Vemde^22^; Tiny Simons^30^; Gert Messchendorp^28^; Nynke Nicolaij^28^; Hester Bongenaar^32^; Karin Bodde^24^; Sandra Kleijn^34^; Jasmijn Lodico^34^; Hanneke Droste^34^; Maureen Wollaert^5^; Sabrina Verheesen^5^; D. Jeurrissen^5^; Erna Bos^9^; Yvonne Drabbe^15^; Michelle Sandiman^15^; Nicoline Aaldering^11^; Berber Zweedijk^17^; Jocova Vervoort^21^; Eva Ponjee^22^; Sharon Romviel^19^; Karin Kanselaar^19^; Denn Barning^10^ ; Laurine van der Steen^3^.

Clinical/imaging data aquisition

Esmee Venema^40^; Vicky Chalos^1,40^; Ralph R. Geuskens^3^; Tim van Straaten^19^; Saliha Ergezen^1^; Roger R.M. Harmsma^1^; Daan Muijres^1^; Anouk de Jong^1^; Olvert A. Berkhemer^1,3,6^; Anna M.M. Boers^3,39^; J. Huguet^3^; P.F.C. Groot^3^; Marieke A. Mens^3^; Katinka R. van Kranendonk^3^; Kilian M. Treurniet^3^; Manon L. Tolhuisen^3,39^; Heitor Alves^3^; Annick J. Weterings^3^; Eleonora L.F. Kirkels^3^; Eva J.H.F. Voogd^11^; Lieve M. Schupp^3^; Sabine L. Collette^28,29^; Adrien E.D. Groot^4^; Natalie E. LeCouffe^4^; Praneeta R. Konduri^39^; Haryadi Prasetya^39^; Nerea Arrarte-Terreros^39^; Lucas A. Ramos^39^ ; Nikki Boodt^1,2,40^; Anne F.A.V Pirson^5^; Agnetha A.E. Bruggeman^3^; Nadinda A.M. van der Ende ^1,2^, Rabia Deniz^3^, Susanne G.H. Olthuis^5,44^, Floor Pinckaers^6,44^

List of affiliations

Department of Neurology^1^, Radiology^2^, Public Health^40^, Erasmus MC University Medical Center;

Department of Radiology and Nuclear Medicine^3^, Neurology^4^, Biomedical Engineering & Physics^39^, Amsterdam UMC, location University of Amsterdam;

Department of Neurology^5^, Radiology & Nuclear Medicine^6^, Maastricht University Medical Center+; School for Cardiovascular Diseases Maastricht (CARIM)^44^; and MHeNs School for Mental Health and Neuroscience, Maastricht, the Netherlands^45^;Department of Neurology^7^, Radiology^8^, Sint Antonius Hospital, Nieuwegein;

Department of Neurology^9^, Radiology^10^, Leiden University Medical Center;

Department of Neurology^11^, Radiology^12^, Rijnstate Hospital, Arnhem; Department of Radiology^13^, Neurology^14^, Haaglanden MC, the Hague;

Department of Neurology^15^, Radiology^16^, HAGA Hospital, the Hague; Department of Neurology^17^, Radiology^18^, University Medical Center Utrecht; Department of Neurology^19^, Neurosurgery^20^, Radiology^27^, Radboud University Medical Center, Nijmegen;

Department of Neurology^21^, Radiology^26^, Elisabeth-TweeSteden ziekenhuis, Tilburg;

Department of Neurology^22^, Radiology^23^, Isala Klinieken, Zwolle;

Department of Neurology^24^, Radiology^25^, Reinier de Graaf Gasthuis, Delft;

Department of Neurology^28^, Radiology^29^, University Medical Center Groningen;

Department of Neurology^30^, Radiology^31^, Zuyderland Medical Center, Heerlen;

Department of Neurology^32^, Radiology^33^, Catharina Hospital, Eindhoven;

Department of Neurology^34^, Radiology^35^, Medisch Spectrum Twente, Enschede, (currently Deventer Hospital^47^);

Department of Radiology^36^, Amsterdam UMC, Vrije Universiteit van Amsterdam, Amsterdam;

Department of Radiology^37^, Noordwest Ziekenhuisgroep, Alkmaar;

Department of Radiology^38^, Texas Stroke Institute, Texas, United States of America;

Department of Neurology^42^, Radiology^41^, Albert Schweitzer Hospital, Dordrecht;

Department of Neurology^43^, Radiology^46^, Amphia Hospital, Breda.
